# Supplementary material for: Role and mechanism of NCAPD3 in promoting malignant behaviors in gastric cancer
Source: Front Pharmacol. 2024 Apr 22;15:1341039. doi: 10.3389/fphar.2024.1341039 (PMC11070777; doi:10.3389/fphar.2024.1341039)
Supplement: Supplementary file 11 [file DataSheet2.ZIP › GSEA/GO_CC/my_analysis.Gsea.1599461408869/gsea_report_for_NCAPD3_MUT_1599461408869.html]

Report for NCAPD3\_MUT 1599461408869 [GSEA]

| GS  follow link to MSigDB | GS DETAILS | SIZE | ES | NES | NOM p-val | FDR q-val | FWER p-val | RANK AT MAX | LEADING EDGE || 1 | GO\_CELL\_SURFACE | Details ... | 61 | -0.35 | -2.39 | 0.000 | 0.007 | 0.005 | 599 | tags=79%, list=42%, signal=131% |
| 2 | GO\_CONTRACTILE\_FIBER | Details ... | 20 | -0.49 | -2.25 | 0.003 | 0.015 | 0.023 | 164 | tags=50%, list=12%, signal=56% |
| 3 | GO\_SIDE\_OF\_MEMBRANE | Details ... | 39 | -0.35 | -2.01 | 0.000 | 0.054 | 0.108 | 589 | tags=77%, list=42%, signal=128% |
| 4 | GO\_EXTRACELLULAR\_MATRIX | Details ... | 34 | -0.36 | -1.99 | 0.002 | 0.043 | 0.115 | 356 | tags=56%, list=25%, signal=73% |
| 5 | GO\_SUPRAMOLECULAR\_POLYMER | Details ... | 72 | -0.28 | -1.97 | 0.000 | 0.039 | 0.131 | 164 | tags=29%, list=12%, signal=31% |
| 6 | GO\_ACTIN\_BASED\_CELL\_PROJECTION | Details ... | 19 | -0.42 | -1.87 | 0.012 | 0.065 | 0.256 | 221 | tags=42%, list=16%, signal=49% |
| 7 | GO\_EXTERNAL\_SIDE\_OF\_PLASMA\_MEMBRANE | Details ... | 25 | -0.36 | -1.85 | 0.002 | 0.063 | 0.282 | 589 | tags=80%, list=42%, signal=135% |
| 8 | GO\_SUPRAMOLECULAR\_COMPLEX | Details ... | 98 | -0.23 | -1.73 | 0.003 | 0.112 | 0.486 | 164 | tags=24%, list=12%, signal=26% |
| 9 | GO\_SPECIFIC\_GRANULE | Details ... | 16 | -0.41 | -1.72 | 0.029 | 0.109 | 0.514 | 468 | tags=69%, list=33%, signal=102% |
| 10 | GO\_PERINUCLEAR\_REGION\_OF\_CYTOPLASM | Details ... | 60 | -0.26 | -1.72 | 0.006 | 0.099 | 0.517 | 359 | tags=43%, list=25%, signal=56% |
| 11 | GO\_ENDOPLASMIC\_RETICULUM | Details ... | 123 | -0.21 | -1.68 | 0.012 | 0.114 | 0.602 | 227 | tags=28%, list=16%, signal=30% |
| 12 | GO\_SYNAPSE | Details ... | 78 | -0.23 | -1.67 | 0.021 | 0.108 | 0.617 | 191 | tags=28%, list=14%, signal=31% |
| 13 | GO\_ADHERENS\_JUNCTION | Details ... | 25 | -0.33 | -1.65 | 0.024 | 0.112 | 0.665 | 263 | tags=44%, list=19%, signal=53% |
| 14 | GO\_CELL | Details ... | 70 | -0.23 | -1.62 | 0.011 | 0.119 | 0.709 | 219 | tags=27%, list=16%, signal=31% |
| 15 | GO\_MEMBRANE\_REGION | Details ... | 24 | -0.32 | -1.58 | 0.042 | 0.135 | 0.779 | 468 | tags=58%, list=33%, signal=86% |
| 16 | GO\_GLUTAMATERGIC\_SYNAPSE | Details ... | 22 | -0.33 | -1.58 | 0.050 | 0.130 | 0.790 | 112 | tags=27%, list=8%, signal=29% |
| 17 | GO\_VESICLE\_LUMEN | Details ... | 30 | -0.29 | -1.57 | 0.047 | 0.128 | 0.805 | 401 | tags=53%, list=28%, signal=73% |
| 18 | GO\_SECRETORY\_GRANULE | Details ... | 63 | -0.23 | -1.57 | 0.026 | 0.121 | 0.810 | 401 | tags=46%, list=28%, signal=61% |
| 19 | GO\_BASOLATERAL\_PLASMA\_MEMBRANE | Details ... | 23 | -0.32 | -1.57 | 0.044 | 0.118 | 0.818 | 146 | tags=26%, list=10%, signal=29% |
| 20 | GO\_GOLGI\_MEMBRANE | Details ... | 50 | -0.24 | -1.55 | 0.048 | 0.121 | 0.842 | 372 | tags=46%, list=26%, signal=60% |
| 21 | GO\_PLASMA\_MEMBRANE\_REGION |  | 86 | -0.20 | -1.54 | 0.039 | 0.123 | 0.861 | 207 | tags=24%, list=15%, signal=27% |
| 22 | GO\_PRESYNAPSE |  | 17 | -0.35 | -1.50 | 0.057 | 0.147 | 0.915 | 190 | tags=35%, list=13%, signal=40% |
| 23 | GO\_SECRETORY\_VESICLE |  | 65 | -0.21 | -1.47 | 0.050 | 0.161 | 0.938 | 401 | tags=45%, list=28%, signal=59% |
| 24 | GO\_INTERMEDIATE\_FILAMENT\_CYTOSKELETON |  | 15 | -0.34 | -1.42 | 0.092 | 0.198 | 0.966 | 244 | tags=40%, list=17%, signal=48% |
| 25 | GO\_POLYMERIC\_CYTOSKELETAL\_FIBER |  | 57 | -0.21 | -1.38 | 0.111 | 0.233 | 0.988 | 156 | tags=23%, list=11%, signal=25% |
| 26 | GO\_COLLAGEN\_CONTAINING\_EXTRACELLULAR\_MATRIX |  | 26 | -0.27 | -1.36 | 0.099 | 0.244 | 0.990 | 526 | tags=69%, list=37%, signal=108% |
| 27 | GO\_NUCLEAR\_OUTER\_MEMBRANE\_ENDOPLASMIC\_RETICULUM\_MEMBRANE\_NETWORK |  | 88 | -0.18 | -1.35 | 0.088 | 0.248 | 0.994 | 189 | tags=23%, list=13%, signal=25% |
| 28 | GO\_CELL\_CELL\_JUNCTION |  | 52 | -0.21 | -1.34 | 0.097 | 0.249 | 0.995 | 347 | tags=40%, list=25%, signal=52% |
| 29 | GO\_GOLGI\_APPARATUS |  | 114 | -0.17 | -1.33 | 0.087 | 0.252 | 0.997 | 357 | tags=35%, list=25%, signal=43% |
| 30 | GO\_ANCHORING\_JUNCTION |  | 87 | -0.18 | -1.33 | 0.098 | 0.243 | 0.997 | 555 | tags=59%, list=39%, signal=91% |
| 31 | GO\_VACUOLE |  | 62 | -0.19 | -1.30 | 0.126 | 0.277 | 0.999 | 273 | tags=27%, list=19%, signal=33% |
| 32 | GO\_ACTIN\_CYTOSKELETON |  | 48 | -0.20 | -1.27 | 0.161 | 0.303 | 1.000 | 223 | tags=27%, list=16%, signal=31% |
| 33 | GO\_SYNAPTIC\_MEMBRANE |  | 24 | -0.26 | -1.26 | 0.164 | 0.303 | 1.000 | 190 | tags=29%, list=13%, signal=33% |
| 34 | GO\_AXON |  | 45 | -0.20 | -1.25 | 0.168 | 0.307 | 1.000 | 184 | tags=24%, list=13%, signal=27% |
| 35 | GO\_ENDOPLASMIC\_RETICULUM\_LUMEN |  | 20 | -0.26 | -1.20 | 0.238 | 0.372 | 1.000 | 493 | tags=65%, list=35%, signal=98% |
| 36 | GO\_CELL\_SUBSTRATE\_JUNCTION |  | 42 | -0.21 | -1.20 | 0.219 | 0.364 | 1.000 | 640 | tags=71%, list=45%, signal=127% |
| 37 | GO\_VESICLE\_MEMBRANE |  | 49 | -0.18 | -1.17 | 0.274 | 0.400 | 1.000 | 505 | tags=53%, list=36%, signal=80% |
| 38 | GO\_SPINDLE\_POLE |  | 17 | -0.25 | -1.09 | 0.335 | 0.512 | 1.000 | 1064 | tags=100%, list=75%, signal=402% |
| 39 | GO\_CELL\_PROJECTION\_MEMBRANE |  | 30 | -0.21 | -1.09 | 0.327 | 0.507 | 1.000 | 207 | tags=27%, list=15%, signal=31% |
| 40 | GO\_CELL\_CORTEX |  | 31 | -0.20 | -1.07 | 0.354 | 0.529 | 1.000 | 476 | tags=52%, list=34%, signal=76% |
| 41 | GO\_CELL\_JUNCTION |  | 109 | -0.13 | -1.04 | 0.403 | 0.570 | 1.000 | 555 | tags=54%, list=39%, signal=82% |
| 42 | GO\_SECRETORY\_GRANULE\_MEMBRANE |  | 20 | -0.23 | -1.03 | 0.399 | 0.580 | 1.000 | 468 | tags=55%, list=33%, signal=81% |
| 43 | GO\_RECEPTOR\_COMPLEX |  | 28 | -0.20 | -1.01 | 0.447 | 0.590 | 1.000 | 54 | tags=14%, list=4%, signal=15% |
| 44 | GO\_COATED\_VESICLE |  | 17 | -0.24 | -1.01 | 0.435 | 0.579 | 1.000 | 372 | tags=41%, list=26%, signal=55% |
| 45 | GO\_ENDOSOME |  | 75 | -0.14 | -1.01 | 0.464 | 0.570 | 1.000 | 206 | tags=21%, list=15%, signal=24% |
| 46 | GO\_EARLY\_ENDOSOME |  | 37 | -0.16 | -0.96 | 0.526 | 0.642 | 1.000 | 191 | tags=22%, list=14%, signal=24% |
| 47 | GO\_EXTRINSIC\_COMPONENT\_OF\_MEMBRANE |  | 29 | -0.18 | -0.95 | 0.541 | 0.656 | 1.000 | 505 | tags=52%, list=36%, signal=79% |
| 48 | GO\_CHROMATIN |  | 103 | -0.12 | -0.94 | 0.586 | 0.652 | 1.000 | 209 | tags=19%, list=15%, signal=21% |
| 49 | GO\_INTRINSIC\_COMPONENT\_OF\_ORGANELLE\_MEMBRANE |  | 25 | -0.19 | -0.94 | 0.547 | 0.649 | 1.000 | 1150 | tags=100%, list=82%, signal=531% |
| 50 | GO\_MICROTUBULE |  | 39 | -0.15 | -0.86 | 0.669 | 0.759 | 1.000 | 156 | tags=18%, list=11%, signal=20% |
| 51 | GO\_ORGANELLE\_SUBCOMPARTMENT |  | 26 | -0.16 | -0.82 | 0.689 | 0.807 | 1.000 | 201 | tags=23%, list=14%, signal=26% |
| 52 | GO\_TRANS\_GOLGI\_NETWORK |  | 15 | -0.19 | -0.79 | 0.702 | 0.839 | 1.000 | 201 | tags=27%, list=14%, signal=31% |
| 53 | GO\_ENDOCYTIC\_VESICLE |  | 15 | -0.19 | -0.77 | 0.772 | 0.856 | 1.000 | 329 | tags=40%, list=23%, signal=52% |
| 54 | GO\_MEMBRANE\_PROTEIN\_COMPLEX |  | 66 | -0.11 | -0.73 | 0.868 | 0.890 | 1.000 | 175 | tags=15%, list=12%, signal=16% |
| 55 | GO\_POSTSYNAPTIC\_MEMBRANE |  | 17 | -0.17 | -0.72 | 0.824 | 0.882 | 1.000 | 184 | tags=24%, list=13%, signal=27% |
| 56 | GO\_NUCLEAR\_CHROMOSOME |  | 99 | -0.09 | -0.72 | 0.882 | 0.871 | 1.000 | 245 | tags=20%, list=17%, signal=23% |
| 57 | GO\_LEADING\_EDGE\_MEMBRANE |  | 16 | -0.17 | -0.70 | 0.846 | 0.873 | 1.000 | 510 | tags=56%, list=36%, signal=87% |
| 58 | GO\_SPINDLE |  | 28 | -0.12 | -0.60 | 0.924 | 0.941 | 1.000 | 312 | tags=29%, list=22%, signal=36% |
Table: Gene sets enriched in phenotype **NCAPD3\_MUT (3 samples) **[plain text format]****
